# Higher concentrations of folic acid reduced the dietary requirements of supplemental methionine for commercial broilers

**DOI:** 10.5713/ab.22.0374

**Published:** 2023-06-26

**Authors:** S. V. Rama Rao, M. V. L. N. Raju, D. Nagalakshmi, T. Srilatha, S. S. Paul, B. Prakash, A. Kannan

**Affiliations:** 1 ICAR - Directorate of Poultry Research, Rajendranagar, Hyderabad 500 030, India; 2 Department of Animal Nutrition, College of Veterinary Science, Rajendranagar, Hyderabad 500 030, India

**Keywords:** Antioxidant Variables, Broiler Chicken, Carcass Variables, DL Methionine, Folic Acid, Immune Responses, Performance

## Abstract

**Objective:**

An experiment was conducted to study the effect of supplementing DL methionine (DL Met) at graded concentrations on performance, carcass variables, immune responses and antioxidant variables in broiler chicken fed folic acid (FA) fortified (4 mg/kg) low-methionine diet.

**Methods:**

A basal diet (BD) without supplemental DL Met, but with higher level (4 mg/kg) of FA and a control diet (CD) with the recommended concentration of methionine (Met) were prepared. The BD was supplemented with DL Met at graded concentrations (0%, 10%, 20%, 30%, 40%, and 50% supplemental DL Met of CD). Each diet was fed *ad libitum* to 10 replicates of 5 broiler male chicks in each from 1 to 42 d of age.

**Results:**

Body weight gain (BWG) reduced, and feed conversion ratio (FCR) increased in broilers fed low-Met BD. At 30% and 20% inclusion of DL met, the BWG and FCR, respectively were similar to those fed the CD. Similarly, supplementation of 10% DL Met to the BD significantly increased ready to cook meat yield and breast meat weight, which were similar to those of the CD fed broilers. Lipid peroxidation reduced, the activity of antioxidant enzymes (GSHPx and GSHRx) in serum increased and lymphocyte proliferation increased with increased supplemental DL Met level in the BD. The concentrations of total protein and albumin in serum increased with DL Met supplementation to the BD.

**Conclusion:**

Based on the data, it can be concluded that supplemental Met can be reduced to less than 50% in broiler chicken diets (4.40, 3.94, and 3.39 g/kg, respectively in pre-starter, starter and finisher phases) containing 4 mg/kg FA.

## INTRODUCTION

Methionine (Met) is an essential amino acid, which is required for protein synthesis, immune modulation [1,2] and glutathione synthesis [3], and is involved in more than 100 transmethylation reactions [4]. Methyl donors (MDs) like choline, folic acid (FA) and betaine are known to spare Met by participation in MD function of the amino acid [5–9]. Thus, additional supplementation of diet with these MD would reduce the dietary requirement of Met. Folic acid is an important water soluble vitamin, which plays a pivotal role in the single carbon transfer in the trans-methylation reaction [10]. The FA, in the form of tetrahydrofolate, donates methyl group to homocysteine to synthesize Met in the biological system [11]. Therefore, by supplementing adequate concentrations of FA, the Met drain out for methylation could be minimized. Literature suggested dietary requirement of FA as 2 to 4 mg/kg for optimum performance in chicken [12,13]. Besides methyl donation, supplementation of FA was reported to elicit a positive response in the immune system [14,15] and reduction of oxidative stress [16,17] in the liver of rats.

The data from our laboratory [8,9] indicated improved broiler performance with supplementation of higher concentrations of FA in diets containing sub-optimal concentrations of Met. Supplementation of 4 to 6 mg of FA/kg diet significantly improved the weight gain and FE in broilers fed corn soybean meal diet without crystalline Met, which suggests the sparing function of the FA for the amino acid in the diet. Therefore, it was presumed that Met concentration can be reduced in diets containing higher concentrations of FA. As the cost of FA is lower than DL Met per unit of broiler feed, an attempt was made to reduce the requirement of supplemental DL Met in broiler diet having higher concentrations of FA (4 mg/kg).

## MATERIALS AND METHODS

### Birds and management

Broiler (Vencobb 400) male chicks (n = 350) were randomly and equally distributed into 7 dietary groups having 10 replicates with 5 chicks per replicate. The birds were reared in 3-tier battery brooder pens (0.61×0.75×0.432 m) with wire floor from 1 to 42 d of age in an open sided poultry house. Supplemental heat was provided with incandescent bulbs and coal burner to brood the chicks at 36°C±1°C up to 7 d of age, which was gradually reduced to 28°C±1°C by 21 d of age, after which, the broilers were maintained at ambient temperature (23.4°C to 30.1°C). Fluorescent bulbs were used to provide 20 h light and 4 h dark in a d from d 22 till the end of the experiment. Birds were vaccinated against Newcastle (Lasota) disease at 5 and 21 d and against infectious bursal disease at 11 and 28 d of age. The experiment was conducted by following the guidelines of the Institute Animal Ethics Committee (IAEC/DPR/17/1 dated 1st October 2017).

### Diets

Yellow maize and soybean meal-based basal diets (BD) having 2,925, 3,050, and 3,100 kcal/kg ME and 220, 200, and 189 g crude protein/kg, respectively for pre-starter (1 to 7 d), starter (8 to 21 d), and finisher (22 to 42 d of age) phases were prepared (Table 1). The BD has all the nutrients recommended for the strain (Vencobb 400 Broiler Management Guide; Venkateswara Hatcheries Pvt Ltd, Pune, India) except Met and FA. The BD was supplemented with commercial broiler vitamin premix having all the vitamins, except FA at the recommend concentration. A set of control diets (CD) were prepared by supplementing crystalline Met (DL-Met) to the BD to meet the 100% recommendations of the Met for the strain i.e. 5.52, 4.85, and 3.93 g/kg, respectively in pre-starter, starter and finisher diets. Concentrations of Met in the pre-starter, starter and finisher BD were 3.28, 3.03, and 2.85 g/kg, Met, respectively in pre-starter, starter and finisher diets. The BD was supplemented with 4 mg/kg FA (Badische Anilin- und Soda Fabrik). The test diets were formulated by supplementing the FA-rich BD with crystalline DL-Met at 10%, 20%, 30%, 40%, and 50% concentration of DL-Met supplemented in the CD. Each diet was allotted to 10 replicates of 5 chicks each by following the completely randomized design and fed *ad libitum* from one to 42 d of age. The FA concentration in the premix was estimated using UV spectrophotometric method and distilled water as the blank at an absorbance of 281 nm [18]. The Met concentration in diets was estimated as per Llames and Fontaine [19].

### Parameters recorded

#### Performance parameters

Body weight and feed intake (FI) per replicate were recorded at 21 and 42 d of age. FI per unit body weight gain (BWG) was calculated as the feed conversion ratio (FCR). The body weight of dead birds was considered to calculate the FCR.

#### Carcass variables

Carcass variables were recorded at 43 d of age by slaughtering one bird by cervical dislocation from each replicate having the body weight nearest to the average of the replicate (+3%). Relative weights of ready-to-cook yield (without liver, gizzard, and heart), abdominal fat and breast meat were recorded and expressed as g/kg pre-slaughter live weight.

#### Serum protein fraction

Blood samples (about 2.5 to 3 mL) were collected from the brachial vein of one bird in each replicate at 42 d of age. Diagnostic kits (Product No 72111 and 72131, respectively, M/S Qualigens India, Mumbai, India) were used to calculate the concentration of total protein and albumin in serum.

#### Serum oxidative parameters

The oxidative parameters like lipid peroxidation (LP) and the activities of antioxidant enzymes like red blood cells catalase (RBCC), glutathione peroxidase (GSHPx) and glutathione reductase (GSHRx) in blood were measured. About two mL of blood from one bird per replicate at 43 d of age was collected into a centrifuge tube containing citrate buffer (1.5 mL/10 mL blood) for erythrocyte separation and antioxidant enzyme estimation. The blood samples were centrifuged at 500×g for 15 min at 4°C to separate buffy coat (white blood cells) and form an erythrocyte pellet. The erythrocytes were washed thrice with phosphate buffer solution (pH 7.4). The packed RBC obtained was mixed with an equal volume of phosphate buffered saline and then diluted as per the requirement with distilled water.

The LP was estimated in serum by quantifying malonyl dialdehyde (MDA). The MDA reacts with 2-thiobarbituric acid to form a trimethine colored substance (pink chromogen), which was extracted into butanol. The color intensity was measured at 548 nm. The LP activity in the erythrocytes was expressed in nmol MDA/mg protein [20].

The enzyme catalase decomposes H_2_O_2_ and the rate of decomposition as measured in terms of reduction in absorbance is indicative of the enzyme activity in the serum sample [21]. The activity of GSHPx and GSHRx were estimated following the method of Paglia and Valantine [22].

#### Immune responses

The effect of supplementing graded concentrations of DL Met on cell mediated immunity (CMI) (*in vitro* lymphocyte proliferation ratio [LPR]) and humoral immunity (HI) (antibody response against Newcastle disease [ND] vaccine) were studied. Blood samples were collected at 20 d of age from one bird per replicate in all the treatments to study the CMI and HI responses.

The difference between the *in vitro* proliferation of lymphocytes with and without the stimulant (concanavalin A, Con A) was expressed as the ratio. The LPR was assayed using MTT tetrazodium salt (3-(4,5-dimethylthiazol-2-yl)-2, 5-diphenyl tetrazolium bromide) [23]. About 2 mL of blood was collected from the brachial vein of the bird in a centrifuge tube containing heparin disodium salt (5 mg). One bird from each replicate was used to collect blood samples at 20 d of age. The un-clotted blood sample was layered gently over histopaque 1077 (Sigma, Mumbai, India) and centrifuged at 500×g for 20 minutes at 4°C. The cellular band at the interface was collected and transferred to another tube and washed 3 times with RPMI 1640 medium (AL 028A; Himedia, Mumbai, India). The viable cells were counted by using the trypan blue dye exclusion method and the cell concentration was adjusted to 1×10^7^ cells/mL of RPMI 1640 medium. These cells (10^5^ purified lymphocytes) were used to measure lymphocyte proliferation by adding 10 μL of suspension to each well of a 96-well flat bottom sterile tissue culture plate. Con A (0.9 μg in 150 μL RPMI/well) was used as the stimulant for lymphocyte proliferation. The plate was incubated at 37°C and 5% CO_2_ concentration for 69 h in a humid atmosphere, then 20 μL MTT (10 mg/mL) was added to each well and the plate was re-incubated for 3 h. At 72 h, 100 μL of 4% 1 N HCl - isopropanol was added to each well and mixed thoroughly with a micropipette to dissolve the formazin crystals, which gave a deep purple colour. The colour intensity was measured in an ELISA reader (V.200.1, μ Quant; Biotek Instruments, Inc., Winooski, VT, USA) at 550 nm. The LPR was calculated as (optical density [OD] of well with Con A – OD of well without Con A)/OD of well without Con A.

The broilers were vaccinated against ND by ocular route at 5 and 21 d of age with Lasota strain (ND Lasota Vac-500; Indovax Pvt., Ltd., Hyderabad, India). At 20 d of age, 2 mL of blood was collected from one bird per replicate and the antibody titres in sera against ND vaccine were measured [24] by haemagglutination test.

### Economics

Feed cost to produce kg BWG in different treatment groups was calculated based on the prevailing market price of major feed ingredients, feed additives, DL Met, and FA. Feed intake, BWG, and feed cost of different treatments were considered to calculated the feed cost per unit live weight gain.

### Statistical analysis

The replicate mean was considered as the experimental unit for performance variables, while the data of each bird were considered as the experimental unit for slaughter, immune responses, serum analysis and antioxidant variables. The data were analysed by one-way analysis of variance to test the effect of dietary treatment on various dependant variables studied in the experiment [25]. The treatment means were compared with Tukey’s test at p<0.05. The effect of supplementing graded concentrations of Met to the BD on various dependant variables were tested with a regression equation Y = a+bx+cx^2^, where Y is the dependant variable and x is the concentration of supplemental DL met in the diet.

## RESULTS

The FA content in the pre-starter, starter and finisher BDs were respectively 4.82, 4.72, and 4.69 mg/kg. Similarly, the analysed concentrations of Met and lysine in the respective diets were 3.21 and 11.4, 2.97, and 10.1, and 2.80 and 9.10 g/kg.

### Performance

The BWG and FCR at d 21 were not affected (p>0.05) by the treatments employed in the current study (Table 2). At the end of the experiment (d 42), the BWG (p = 0.024) reduced and FCR (p = 0.001) increased in broilers fed low-Met BD. The weight gain increased progressively with DL Met supplementation and at 30% inclusion, the BWG was similar to those fed the CD. Similarly, at 20% inclusion of Met to the BD, the FCR reduced similar to the level of the CD. And a further increase in DL Met did not show any additional influence on these performance variables.

The regression analysis indicated that the performance variables were not affected (p>0.05) by DL Met supplementation to the BD at day 21, while both BWG and FCR increased (p = 0.011) and reduced (p = 0.001), respectively in a non–linear manner, with an increase in DL Met concentration in the BD.

The feed cost required to gain kg live weight in the BD fed broilers was 0.73 USD which was 7 cents higher than those fed the CD diet (0.66 USD). The feed cost in broilers fed 40% DL Met and 50% DL Met was similar to those fed the CD (0.66 USD).

### Slaughter variables

The ready to cook (RTC) yield reduced and abdominal fat content increased in broilers fed the low Met BD compared to the CD group (Table 3). Supplementation of 10% DL Met to the BD increased (p = 0.001) the RTC yield, which was like the CD fed broilers. Similarly, the abdominal fat content reduced (p = 0.008) progressively with DL Met supplementation and the fat deposition at 30% or 40% DL Met was significantly higher than the BD, but similar to the CD groups. The relative weight of breast meat weight at 40% DL Met supplementation to the BD was higher (p = 0.023) than those fed the BD without supplemental DL Met. However, the breast weight in DL Met supplementation at all levels was similar to the CD group. The relative weights of giblet and bursa were not affected with the dietary variations. The lowest weight of spleen was recorded in broilers fed the BD, but supplementation of DL Met increased the organ weight and at 50% inclusion, the spleen weight was significantly higher than the BD and the organ weight in other groups was similar to the CD group.

The relative weights of RTC (p = 0.001), breast meat (p = 0.032), and spleen (p = 0.067) increased non-linearly, while the abdominal fat (p = 0.011) decreased non-linearly with increased DL Met concentration in the BD.

### Antioxidant variables

Though the LP reduced in the BD-fed broilers compared to the CD group, the difference between these groups was not statistically significant (Table 4). However, the LP reduced progressively with increase in the supplemental DL Met and the LP was reduced (p = 0.001) at 20% or higher Met supplementation to the BD compared to the CD group. The lowest LP was observed at the highest DL Met inclusion in the BD. The activity of both the antioxidant enzymes (GSHPx and GSHRx) was reduced (p = 0.001) in the low-Met BD fed broilers compared to those fed the CD diet. In general, supplementation of DL Met increased the activity of both the enzymes. Maximum activity of GSHRx and GSHPx were observed, respectively at 50% and 40% inclusion of DL Met in the BD.

Similarly, the regression analysis indicated a non-linear decrease in LP (p = 0.001) and increase in the activities of GSHPx (p = 0.001) and GSHRx (p = 0.001) in serum of broilers fed higher concentration of DL Met in the BD.

### Immune responses

The antibody titre against ND vaccine was not affected (p< 0.05) by the treatments employed in the current study (Table 4). The cell mediated immune response in terms of LPR was reduced (p = 0.001) in broilers fed the BD compared to those fed the CD. In general, the LPR increased with the level of supplemental DL met and at 50% amino acid inclusion, the LPR was higher than those fed the CD. Increase in concentration of the DL Met in the BD non-linearly (p = 0.001) increased the LPR, however such improvement was not observed (p>0.05) in antibody titre against ND vaccine.

### Serum protein fractions

The serum total protein was significantly (p<0.05) reduced in the BD fed broilers compared to the CD group (Table 4). Both total protein and albumin concentrations increased (p = 0.001) with the inclusion of 20% DL Met compared to the BD. Higher inclusion levels (>20%) did not show any additional improvement in the concentrations of these protein fractions. The regression analysis indicated significant and non-linear increase (p = 0.001) in the concentrations of total protein and albumin with increase in DL Met concentration in the diet. The serum cholesterol concentration reduced non-linearly (p<0.001) with an increase in DL Met in the BD.

## DISCUSSION

Compared to the CD, feeding low-Met BD resulted in a significant reduction in BWG and feed efficiency (FE) in broilers at d 42. This implies that Met deficiency caused a significant reduction in growth and FE in broiler chicken. Similar to the current study, previous work [5,8,9] also showed a significant reduction in performance of broilers fed maize-soybean meal diets without supplemental Met. The primary hypothesis of the study assumed that at higher inclusion levels of FA, the dietary requirement of DL Met will be reduced due to the sparing effect of additional dietary levels of FA on the MD function of Met. As expected, the BWG at 30% and FE at 20% inclusion of the DL Met to FA fortified BD was significantly improved over the BD and were similar to those fed the CD having 100% DL Met at the end of the experiment. The improved performance of broilers with lower inclusion levels of DL Met to the BD (FA fortified) suggested the reduced requirement of DL Met in diets containing higher levels of FA. The reduced requirement of Met in FA-fortified BD can be explained by the role of FA in single carbon transfer reactions. As the FA is the nucleolus in the transmethylation cycle, inclusion of higher concentrations of FA and betaine were reported to spare Met from the MD function [26,2]. Similarly, in our previous studies, inclusion of higher levels of FA or other MDs (betaine, biotin, vitamin B_12_) improved the performance (BWG and or FE) of broilers fed Met-deficient maize-soybean meal-based diets [7–9]. The higher levels of FA were able to support the broiler performance when supplemented to the diets with sub-optimal levels of Met. The improvement in broiler performance with the addition of DL Met was reported to be mediated by stimulating synthesis and release of insulin-like growth factor I [27] with Met supplementation. Though the cost of feed ingredients and additives are highly variable, the current study indicated that the feed cost per kg BWG in broilers fed the BD with 40% or 50% DL Met compared to those fed the CD. These observations imply that the possibility of sparing about 50% of supplemental DL met in broiler diet without affecting the performance and feed cost per kg BWG.

The reduced requirement of supplemental DL Met (or dietary requirement of the amino acid) in the presence of higher levels of FA could be due to the role of FA in the conversion of homocysteine to Met by donating methyl group through transmethylation. Similarly, the Met sparing function of certain MDs (FA, choline, or DL-Met) was reported to improve the growth in broiler chicks fed the diets having lower concentrations (<3.8 g/kg) of Met [28,29].

Reduction in RTC yield and breast weight in broilers fed the low-Met BD compared to the CD indicate that the levels of Met in the BD fortified with FA appears to be inadequate to support these carcass variables. However, supplementation of 10% and 40% DL Met to the BD, respectively improved the RTC yield and breast meat weight similar to the Met-adequate CD fed broilers. These results of carcass variables clearly indicated that the presence of FA could considerably reduce the requirement of supplemental DL Met to a level lower than those present in the CD. In line with results of the current study, the recent finding of Akter et al [30] reported significant improvement in dressing percent and the relative weight of the back in broilers fed 0.3% supplemental L Met in the diet. Similar improvement in the breast meat yield was reported with supplementation of certain MDs (betaine or vitamin B12) to diets either with adequate Met or its sub-optimal concentration in the diet [4,26]. Similarly, Acar et al [31] reported reduced abdominal fat deposition in broilers fed higher dietary Met. The increase in abdominal fat deposition in the BD-fed broilers could be due to inadequate concentration of Met in the diet. The lower dietary Met concentration might have reduced the weight gain probably due to the reduced protein synthesis and thereby favoured the diversion of nutrients to fatty acid synthesis. In the current study, supplementation of even marginal levels of DL Met (30% to 40%) to FA-fortified BD were able to reduce the fat deposition in the abdominal area. As the level of DL Met increased, the BWG improved (and more protein accretion), which might have reduced the fatty acid synthesis and abdominal fat deposition.

Though the weight of bursa was not affected, the relative weight of spleen increased progressively with the levels of DL Met in the diet. The increase in the lymphoid organ weight was also reflected in a significant improvement in the cell mediated immune response (LPR) at 20% to 50% DL Met in diet compared to the BD fed broilers. The improved LPR observed at lower inclusion levels of DL Met (50%) compared to the CD (100% supplemental Met) could be due to the possible synthesis of Met from homocysteine in the presence of FA (present in the BD), which might have increased the availability of Met, whose immune modulator role is well established [32,33,2]. Similar to these findings, an increase in CMI response to PHA-P inoculation was observed in our previous study with increased concentrations of Met in broiler diet [2]. Similarly, a higher immune response in broiler chickens was reported when they were fed higher Met levels in the diet [34,35].

Variations in environmental temperature and humidity in open type poultry houses, which are common in tropical countries, will have a direct impact on the bird’s thermoregulation and make them prone to oxidative stress. The thermal stress leads to oxidation processes and excess production of free radicals, which are scavenged by anti-oxidant enzymes like GSHPx and GSHRx and converted into less harmful substances. In the current study, the LP was not affected, but the activities of GSHPx and GSHRx were reduced in broilers fed the low-Met BD compared to those fed the CD. The reduced activities of antioxidant enzymes in broilers fed the BD could be due to the deficit levels of Met, which is essential for synthesis of glutathione. Similarly, Met is known to increase the expression levels of genes related to antioxidant activity, particularly under heat stress conditions [36]. The oxidative stress appears to be reduced (progressive and significant reduction in LP and increase in activities of GSHPx and GSHRx) in broilers fed with higher levels of DL Met in the BD. Similar to the current findings, our previous studies [8,9] also indicated the reduced stress indices and improved immune responses in broilers fed MDs like FA, biotin, betaine and vitamin B12.

The increased broiler performance in the current study with DL Met supplementation to BD is likely due to improved protein synthesis [37,38]. In general, the concentrations of total protein and albumin in serum increased with supplementing DL Met to the BD. The concentrations of these protein fractions at higher levels of supplemental Met were higher than those fed the BD and equal or higher than those fed the CD. The increased concentration of protein fractions in serum suggested an increased protein synthesis by supplementing DL Met to the BD. Similar improvement in the concentrations of total protein, globulin and albumin in plasma or serum with Met or MD supplementation was also recorded in our previous studies [8,9].

## CONCLUSION

Based on the data of the current study, it can be concluded that the dietary requirement of supplemental Met for broilers can be reduced to 50% in diets (4.40, 3.94, and 3.39 g/kg, respectively in pre-starter, starter, and finisher phases) containing 4 mg/kg FA.

## Figures and Tables

**Table 1:** Ingredient and nutrient composition (g/kg) of basal diets

Ingredient	Pre starter (1 to 7 d)	Starter (8 to 21 d)	Finisher (22 to 42 d)
Maize	555.3	597.9	641.5
Soybean meal	387.0	334.0	292.0
Vegetable oil	21.5	33.2	32.8
Common salt	2.50	2.48	2.45
Sodium bi-carbonate	1.00	1.00	1.00
Dicalcium phosphate	18.8	17.5	16.6
Shell grit	10.90	10.92	10.65
DL-methionine	0.0	0.0	0.0
L-lysine HCl	0.0	0.0	0.0
Additives^1)^	3.00	3.00	3.00
Nutrient			
Metabolizable energy (MJ/kg)^2)^	12.24	12.76	12.97
Crude protein^2)^	220	200	185
Crude protein^3)^	226	206	191
Lysine^2)^	12.1	10.8	9.50
Lysine^3)^	11.4	10.1	9.10
Mehionine^2)^	3.28	3.03	2.85
Methionine^3)^	3.21	2.97	2.80
Threonine^2)^	7.97	7.26	6.72
Tryptophan^2)^	2.58	2.29	2.07
Folic acid (mg/kg)^3)^	4.82	4.72	4.69
Calcium^2)^	9.00	8.50	8.00
Non-phytate phosphorus^2)^	4.50	4.20	4.00

1) Provided (mg/kg diet): thiamin 1; pyridoxine, 2; cyanocobalamine, 0.01; niacin, 15; pantothenic acid, 10; a tocopherol, 10; riboflavin, 10; biotin, 0.08; menadione, 2; retinol acetate, 2.75; cholecalciferol, 0.06; choline, 650; copper, 8; iron, 45; manganese, 80; zinc, 60; selenium, 0.18; hydrated sodium calcium alumino silicates, 800; phytase, 375 units

2) Calculated values.

3) Analysed values.

**Table 2:** Performance of broiler chickens fed graded concentrations of supplemental methionine in diets fortified with folic acid

Treat	1 to 21 d		1 to 42 d	
	
	BWG (g)	FI/BWG	BWG (g)	FI/BWG
CD	924.2	1.232	2,534^A^	1.638^B^
BD	881.0	1.271	2,259^B^	1.828^A^
DLM 10^1)^	902.6	1.292	2,378^AB^	1.800^A^
DLM 20^1)^	848.1	1.250	2,375^AB^	1.711^B^
DLM 30^1)^	870.5	1.293	2,493^A^	1.706^B^
DLM 40^1)^	864.8	1.259	2,415^AB^	1.673^B^
DLM 50^1)^	910.4	1.295	2,541^A^	1.681^B^
SEM	8.720	0.0076	25.308	0.0130
p-value				
ANOVA	0.207	0.184	0.024	0.001
Linear	0.773	0.720	0.003	0.001
Quadratic	0.317	0.815	0.011	0.001

BWG body weight gain, FI fed intake, CD control diet; BD basal diet; SEM, standard error mean; p-value, probability; ANOVA, analysis of variance.

1) DLM 10 to 50 supplemental DL methionine concentration 10% to 50% of the CD.

A,B Means having common superscript in a column do not vary significantly (p<0.05).

**Table 3:** Slaughter variables and relative weight of lymphoid organs (g/kg live weight) in broiler chickens fed graded concentrations of supplemental methionine in diets fortified with folic acid

Treat	RTC	Breast meat	Abdominal fat	Giblet	Bursa	Spleen
CD	736.3^A^	237.8^AB^	13.79^C^	47.94	1.902	2.440^AB^
BD	691.4^B^	227.1^B^	19.55^A^	50.18	1.758	1.732^B^
DLM 10^1)^	727.8^A^	239.0^AB^	18.93^AB^	50.68	1.776	1.925^B^
DLM 20^1)^	738.2^A^	242.5^AB^	17.48^ABC^	46.33	1.579	2.174^AB^
DLM 30^1)^	739.4^A^	238.0^AB^	15.69^BC^	51.30	1.770	2.279^AB^
DLM 40^1)^	725.9^A^	252.0^A^	14.28^C^	49.26	1.732	2.201^AB^
DLM 50^1)^	734.2^A^	244.0^AB^	16.75^ABC^	48.32	1.960	2.810^A^
SEM	2.89	2.23	0.5045	0.5152	0.0592	0.0920
p-value						
ANOVA	0.001	0.023	0.008	0.127	0.733	0.047
Linear	0.001	0.017	0.007	0.468	0.362	0.021
Quadratic	0.001	0.022	0.011	0.761	0.277	0.067

RTC, ready to cook, CD control diet; BD basal diet; SEM, standard error mean; p-value, probability; ANOVA, analysis of variance.

1) DLM 10 to 50 supplemental DL methionine concentration 10% to 50% of the CD.

A–C Means having common superscript in a column do not vary significantly (p<0.05).

**Table 4:** Antioxidant variables and immune responses in broiler chickens fed graded concentrations of supplemental methionine in diets fortified with folic acid

Treat	LP (nM MDA/mg protein)	GSHRx	GSHPx	Immune responses		Serum (g/dL)	
		
		Units/mL		LPR	ND titre, log 2	Total protein	Albumin
CD	1.361^A^	76.94^B^	51.83^B^	0.635^B^	4.600	1.735^A^	0.528^C^
BD	1.304^AB^	64.55^E^	46.21^C^	0.414^D^	5.100	0.930^B^	0.533^C^
DLM 10^1)^	1.277^BC^	73.09^BCD^	47.64^C^	0.440^CD^	5.100	0.991^B^	0.548^C^
DLM 20^1)^	1.213^CD^	70.17^CDE^	52.18^B^	0.483^C^	4.100	1.420^A^	0.675^AB^
DLM 30^1)^	1.177^DE^	75.47^BC^	52.18^B^	0.471^C^	5.300	1.460^A^	0.605^BC^
DLM 40^1)^	1.130^E^	68.04^DE^	56.75^A^	0.614^B^	5.300	1.533^A^	0.735^A^
DLM 50^1)^	1.117^E^	91.94^A^	55.22^AB^	0.792^A^	4.400	1.779^A^	0.737^A^
SEM	0.0142	1.261	0.599	0.0167	0.398	0.0558	0.0181
p-value							
ANOVA	0.001	0.001	0..01	0.001	0.280	0.001	0.001
Linear	0.001	0.001	0.001	0.001	0.549	0.001	0.001
Quadratic	0.001	0.001	0.001	0.001	0.826	0.001	0.001

LP, lipid peroxidation; GSHRx, glutathopne reductase; GSHPx, glutathione peroxidase; MDA, malonyl dialdehyde; LPR, lymphocyte proliferation ratio; CD, control diet; BD, basal diet, SEM standard error mean; p-value, probability; ANOVA, analysis of variance.

1) DLM 10 to 50 supplemental DL methionine concentration 10% to 50% of the CD.

A–E Means having common superscript in a column do not vary significantly (p<0.05)

## SUPPLEMENTARY MATERIAL

The data will be provided by the author on request to svramarao1@gmail.com
